# Tracing the Origin of the Fungal α1 Domain Places Its Ancestor in the HMG-Box Superfamily: Implication for Fungal Mating-Type Evolution

**DOI:** 10.1371/journal.pone.0015199

**Published:** 2010-12-08

**Authors:** Tom Martin, Shun-Wen Lu, Herman van Tilbeurgh, Daniel R. Ripoll, Christina Dixelius, B. Gillian Turgeon, Robert Debuchy

**Affiliations:** 1 Department of Plant Biology and Forest Genetics, Uppsala Biocenter, Swedish University of Agricultural Sciences (SLU), Uppsala, Sweden; 2 Department of Plant Pathology and Plant-Microbe Biology, Cornell University, Ithaca, New York, United States of America; 3 Univ Paris-Sud, Institut de Biochimie et de Biophysique Moléculaire et Cellulaire, UMR8619 Univ Paris-Sud CNRS, Orsay, France; 4 Univ Paris-Sud, Institut de Génétique et Microbiologie, UMR8621 Univ Paris-Sud CNRS, Orsay, France; 5 CNRS, Institut de Génétique et Microbiologie, UMR8621 Univ Paris-Sud CNRS, Orsay, France; University College Dublin, Ireland

## Abstract

**Background:**

Fungal mating types in self-incompatible Pezizomycotina are specified by one of two alternate sequences occupying the same locus on corresponding chromosomes. One sequence is characterized by a gene encoding an HMG protein, while the hallmark of the other is a gene encoding a protein with an α1 domain showing similarity to the Matα1p protein of *Saccharomyces cerevisiae*. DNA-binding HMG proteins are ubiquitous and well characterized. In contrast, α1 domain proteins have limited distribution and their evolutionary origin is obscure, precluding a complete understanding of mating-type evolution in Ascomycota. Although much work has focused on the role of the *S. cerevisiae* Matα1p protein as a transcription factor, it has not yet been placed in any of the large families of sequence-specific DNA-binding proteins.

**Methodology/Principal Findings:**

We present sequence comparisons, phylogenetic analyses, and *in silico* predictions of secondary and tertiary structures, which support our hypothesis that the α1 domain is related to the HMG domain. We have also characterized a new conserved motif in α1 proteins of Pezizomycotina. This motif is immediately adjacent to and downstream of the α1 domain and consists of a core sequence Y-[LMIF]-x(3)-G-[WL] embedded in a larger conserved motif.

**Conclusions/Significance:**

Our data suggest that extant α1-box genes originated from an ancestral HMG gene, which confirms the current model of mating-type evolution within the fungal kingdom. We propose to incorporate α1 proteins in a new subclass of HMG proteins termed MATα_HMG.

## Introduction

**Figure 1:**
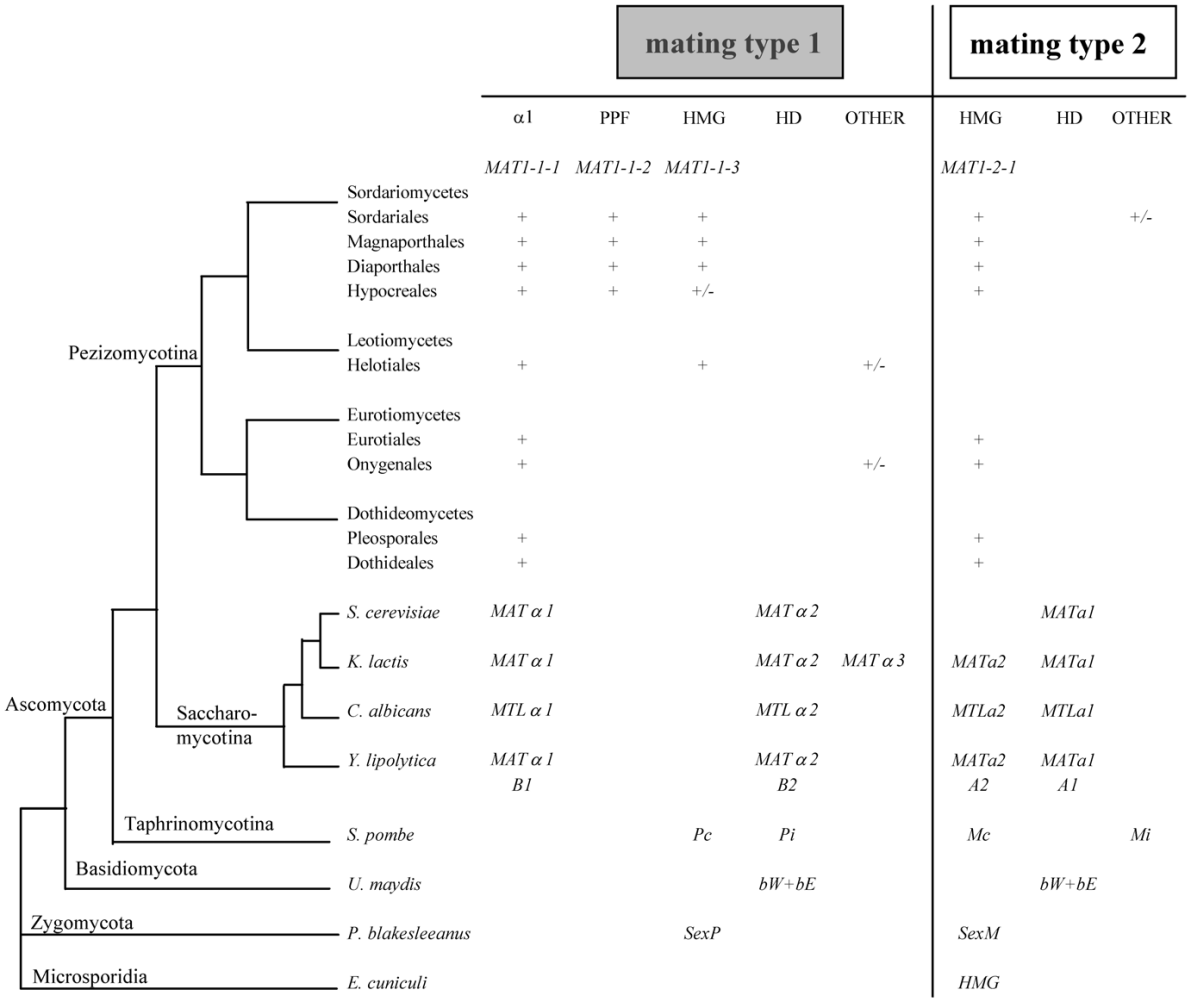
Mating-type structure across the fungal kingdom. α1, genes encoding transcription factors with an α1 domain; PPF, genes encoding proteins with a domain characterized by highly conserved proline and phenylalanine residues [41]; HMG, genes encoding transcription factors with an HMG domain; HD, genes encoding transcription factors with an homeodomain; OTHER, genes encoding proteins not relevant to this study. The standardized nomenclature [7] currently used for Pezizomycotina is indicated below the corresponding domains. +, gene present; +/−, gene present in some species from a group. Mating-type structures were compiled for the following species and corresponding references: *Saccharomyces cerevisiae*, *Kluyveromyces lactis*, *Candida albicans* and *Yarrowia lipolytica* [49], [65], *Schizosaccharomyces pombe* [8], *Ustilago maydis* [66], *Phycomyces blakesleeanus* [17] and *Encephalitozoon cuniculi* [46]. The *Pc* gene from *S*. *pombe* was placed in the HMG class in agreement with the current classification of Pc protein (P10841) in Swissprot. Mating-type genes from *U. maydis, P. blakesleeanus and E. cuniculi* were placed arbitrarily in mating type 1 or 2.

Mating types in fungi display highly variable structure and content (Figure 1); in Ascomycota, they consist of dissimilar sequences occupying the same locus on the chromosome. These sequences are termed idiomorphs, to denote that they are not obviously related by structure or common descent [1]. All mating types are not idiomorphic, and there are examples in Zygomycota and Basidiomycota where they are more accurately considered as conventional alleles [2], [3]. A common feature specific to ascomycotan mating types is the presence in one idiomorph of a gene encoding an α1 protein [3], [4] (Figure 1). The α1 protein Matα1p was initially characterized in *Saccharomyces cerevisiae* [5] and α1 domain proteins were subsequently found to be ubiquitous in Ascomycotina [4], [6]. The constant presence of an α1-box gene in one idiomorph constitutes the basis for mating-type nomenclature in self-incompatible (heterothallic) Pezizomycotina [7]. This gene is called *MAT1-1-1* and defines the *MAT1-1* idiomorph, while the other idiomorph called *MAT1-2,* is characterized by the presence of a *MAT1-2-1* gene which encodes a transcription factor with a MATA_HMG domain. Although no α1 domain was identified in the mating-type P-specific polypeptide Pc of the fission yeast *Schizosaccharomyces pombe* (Taphrinomycotina) when its mating-type proteins were described initially [8], nor in subsequent work [9], limited similarity of the Pc protein to the α1 domain has been reported [9], prompting some authors to speculate that Pc might be an α1-type protein [10]. Currently, Pc is annotated as a HMG protein (*e*.*g*., Swissprot P10841), although neither the HMG nor the α1 classification has been evaluated rigorously in any publication. The exclusive presence of the α1 genes in *MAT* loci of Ascomycota (Pezizomycotina, Saccharomycotina and possibly Taphrinomycotina) prompts questions about mechanisms of acquisition and their ancestry.

In *S. cerevisiae*, Matα1p is a transcriptional co-activator essential for expression of α-specific genes in haploid α cells including those encoding the mating pheromone α-factor and the receptor for the opposite pheromone factor [11]. Matα1p is a pivotal protein which binds cooperatively with the MADS-box transcription factor [12], Mcm1p, and interacts with Ste12p [13] to activate transcription of α-cell specific genes. It has been suggested that the α1 domain may be involved in the physical interaction of Matα1p with Mcm1p [13]. More recently, the α1 domain has been shown to act as a degradation signal, suggesting that rapid turnover of Matα1p is important during yeast mating-type switching [14]. α1 proteins (MAT1-1-1) of Pezizomycotina are also required for mating-type specific transcription of pheromones and pheromone-receptors [4]. Taken together, these lines of evidence support the idea that α1 proteins are transcription factors which bind to DNA via the conserved α1 domain. To our knowledge, however, the relationship of the α1 domain to other DNA-binding domains has not been documented. As a consequence, it has not yet been placed in any of the large families of sequence-specific DNA-binding proteins that are referenced in transcription factor databases (e.g. TRANSFAC [15]) and the α1 domain profile (PDOC51325) in Prosite [16] does not cite a relationship to any well-known DNA binding domain family.

We present sequence comparisons, phylogenetic analyses of mating-type protein domains, and *in silico* predictions of secondary and tertiary structures, which support our hypothesis that the α1 domain is related to the HMG domain. This finding supports the current model for fungal mating-type evolution which links the appearance of the α1 box to a pre-existing HMG box.

## Results and Discussion

### The α1 and the HMG domains share conserved sequences

**Figure 2:**
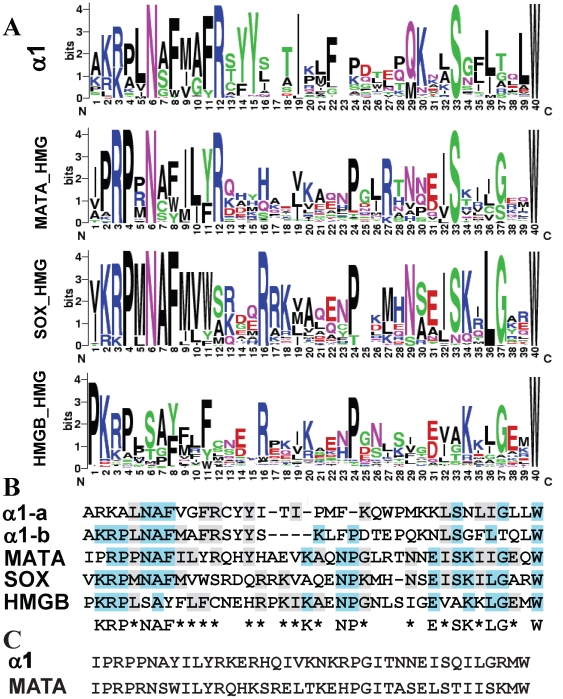
Conserved sequence of α1 and HMG domains. (A). WebLogo [19] representation of conserved sequences in α1, MATA-HMG, SOX and HMGB domains respectively. The x-axis represents amino acid position from the N to C terminal. The amino acid labeled as ‘1’ is located at position 11-48 and 1-2 in the α1 and HMG domains, respectively (NCBI Conserved Domain Database accession numbers: pfam04769 and cd00084). Logos represent an ∼40 amino acid core sequence of the DNA binding domain from 300 α1 domains, and 257 MATA_HMG, 3054 SOX_HMG and 2162 HMGB_UBF_HMG-box domains. (B) Consensus core sequences produced from conserved amino acids in *A*. α1 protein domains divided into those of Pleosporales (α1-a) and Pezizomycotina without Pleosporales (α1-b). α1-a and α1-b are considered as one for identity scoring. Three or more identical amino acids among sequences are coloured blue while two or more identical or similar amino acids are coloured grey. Conservation among the five sequences is shown; a letter is used to represent three or more identical amino acids and an asterisk (*) for two identical or similar amino acids. (C) Ancestral core region for α1 and MATA_HMG. Core regions from 300 α1 domains and 257 MATA_HMG sequences were used.

Certain sequence similarities between MATA_HMG and α1 proteins have been noticed previously [3], [4], [17], however whether this reflects functional analogy was not established. Furthermore, the origin of α1 in HMG has not been explicitly proposed before. Initially, to investigate whether there are similarities between the α1 and MATA_HMG domains, we analyzed a small dataset that included members of each and identified a core region present in both (See Materials and Methods, and Figure S1). Next, a total of 5,773 sequence sets corresponding to α1 domains from Ascomycota and HMG domains from fungi, plants and animals were aligned with the core region using Muscle [18] and conserved sequences identified. Graphical representation of relative frequency of each amino acid derived using WebLogo [19] revealed similarities between HMG and α1 domains, as well as expected similarities among different HMG domain classes. The consensus sequences from the three HMG-domain core regions showed significant similarity. MATA_HMG and SRY-related HMG-box (SOX) [20] had 40% identical amino acids (identity) and 67% identical or similar amino acids (positives) (E value 2e-08), MATA_HMG and HMGB had 36% identity and 65% positives (E value 2e-07), and SOX and HMGB had 35% identity and 61% positives (E value 6e-08). These values would be expected from members of the same domain family. As noted above, strong similarities were also apparent between α1 domains and the HMG domain family (Figure 2A). Alignment of all consensus sequences derived from WebLogo revealed that the α1 domain has features in common with HMG domains (Figure 2B): the α1 and the MATA_HMG consensus sequences were significantly similar (E value 3e-04) with 28% identity and 50% positives. The core α1 domain (α1-a) is two amino acids shorter in Pleosporales and four shorter in all other Pezizomycotina (α1-b) than the core MATA_HMG domain, suggesting that if α1 and HMG domain sequences are indeed evolutionarily related, and if the HMG domain is ancestral, as we argue below, small deletions occurred in the α1 box. The consensus α1 domain showed 32% identity and 45% positives (E value 0.001) with SOX consensus sequences but much less similarity to the HMGB consensus. In that latter case, the alignment program detects only six identical and two positive residues in the first 10 residues (E value 0.011). A hidden Markov Model (HMM) profile-profile test using the α1 dataset and the program COMPASS [21] also identified the HMG domain as the best hit (E value 2.5e-05).

α1 and MATA_HMG domains were used as input for Ancescon [22] to predict ancestral sequences. The predicted ancestral α1 and MATA_HMG sequences (Figure 2C) showed high similarity to each other (E value 6e-11), supporting the hypothesis that they are evolutionary related.

### The α1 domain groups with the MATA_HMG domain group in phylogenetic analyses

**Figure 3:**
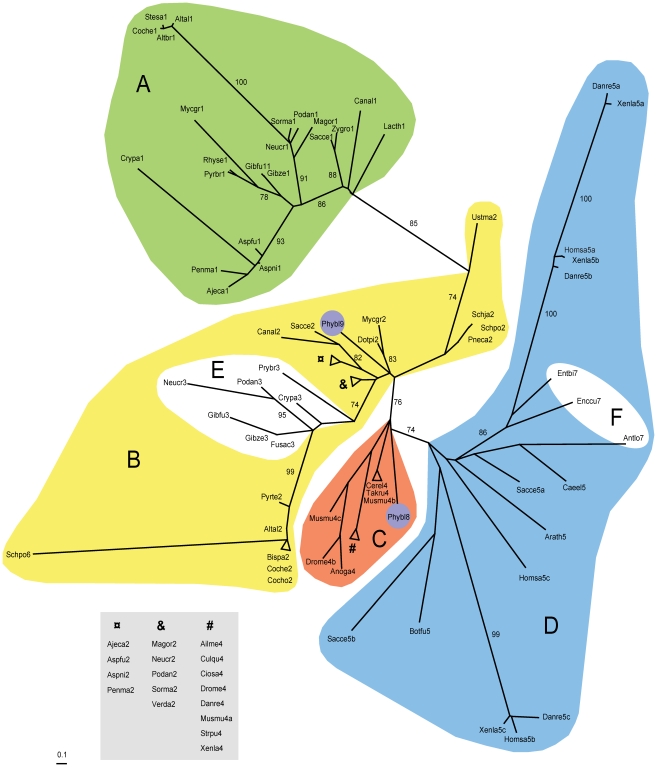
Unrooted phylogram for the HMG superfamily and the α1 domain core amino acid sequences. Clustering of core amino acid sequences using maximum-likelihood and model LG+G [67]. Labelling is as follows: α1 (A, green), MATA_HMG (B, yellow), SOX (C, orange), HMGB (D, blue), MAT1-1-3 subgroup of MATA_HMG (E, white), Microsporidia *MAT* sex locus HMG (F, white), *Phycomyces blakesleeanus* (Zygomycota) sexM (Phybl8) and sexP (Phybl9) are circled in purple. LR-ELW values above 70% are shown. Abbreviations: Ailme, *Ailuropoda melanoleuca;* Ajeca, *Ajellomyces capsulatus;* Altal, *Alternaria alternata;* Altbr, *Alternaria brassicicola;* Anoga, *Anopheles gambiae;* Antlo, *Antonospora locustae;* Arath, *Arabidopsis thaliana;* Aspfu, *Aspergillus fumigatus;* Aspni, *Aspergillus nidulans;* Bipsa, *Bipolaris sacchari;* Botfu, *Botryotinia fuckeliana;* Caee, *Caenorhabditis elegans;* Canal, *Candida albicans;* Cerel, *Cervus elaphus yarkandensis;* Ciosa, *Ciona savignyi;* Coche, *Cochliobolus heterostrophus;* Crypa, *Cryphonectria parasitica;* Culqu, *Culex quinquefasciatus;* Danre, *Danio rerio;* Dotpi, *Dothistroma pini;* Drome, *Drosophila melanogaster;* Enccu, *Encephalitozoon cuniculi;* Entbi, *Enterocytozoon bieneusi;* Fusac, *Fusarium acaciae-mearnsii;* Gibfu, *Gibberella fujikuroi;* Gibze, *Gibberella zeae;* Homsa, *Homo sapiens;* Lacth, *Lachancea thermotolerans;* Magor, *Magnaporthe oryzae;* Musmu, *Mus musculus;* Mycgr, *Mycosphaerella graminicola;* Neucr, *Neurospora crassa;* Penma, *Penicillium marneffei;* Pneca, *Pneumocystis carinii;* Podan, *Podospora anserina;* Pyrbr, *Pyrenopeziza brassicae;* Pyrte, *Pyrenophora teres;* Rhyse, *Rhynchosporium secalis;* Sacce, *Saccharomyces cerevisiae;* Schja, *Schizosaccharomyces japonicus;* Schpo, *Schizosaccharomyces pombe;* Sorma, *Sordaria macrospora;* Stesa, *Stemphylium sarciniforme;* Strpu, *Strongylocentrotus purpuratus;* Takru, *Takifugu rubripes;* Ustma, *Ustilago maydis;* Verda, *Verticillium dahliae;* Xenla, *Xenopus laevis;* Zygro, *Zygosaccharomyces rouxii*. Numbers after species names indicate α1 proteins (1), MATA_HMG (2), MAT1-1-3 (3), SOX (4), HMGB (5) and other HMG domains (6–9). When more than one domain is present for the same species, the suffix a, b or c was added. Accession numbers of species grouped by evolutionary affinity are in Table S1. Units indicate number of amino acid changes per position.

A maximum likelihood phylogram was constructed using a selection of α1 and HMG core domains from representative taxa (Figure 3). LG+G and LG+I+G models [23] were found to best fit the data and produced almost identical phylogenetic trees. The α1 sequences clustered in a monophyletic clade (A in Figure 3) within the MATA_HMG domain sequence branch (B and E in Figure 3) (LR-ELW edge support  = 85). The α1 and MATA_HMG domains clustered separately from SOX (C in Figure 3) and HMGB domains (D in Figure 3) (LR-ELW edge support  = 76). Topology tests [24], [25] also supported the proposed tree (KH *P* = 1, SH *P* = 1). This places the α1 core sequence specifically closer to fungal MATA_HMG sequences than to the other members of the HMG family. The sequence of the putative α1 domain of *S. pombe* Pc (Schpo6) did not group with α1 sequences but instead grouped with the Dothideomycete MATA_HMG sequences with extremely high support (LR-ELW edge support  = 99). Sequences of Sordariomycete and Leotiomycete MAT1-1-3 proteins formed a subgroup (E in Figure 3) within MATA_HMG. The Dothideomycete MATA_HMG sequences were closer to MAT1-1-3 sequences (LR-ELW edge support  = 74) than to MAT1-2-1 sequences. Interestingly, the Zygomycete *P*. *blakesleeanus* sexM (Phybl8) and sexP (Phybl9) sequences grouped with SOX and MATA_HMG, respectively, while the microsporidia sequences (F in Figure 3) grouped with HMGB (D in Figure 3).

Overall these data support the hypothesis that the genes encoding α1 and MATA_HMG proteins are evolutionarily related. The HMG domain is found in all eukaryotes with the HMGB, SOX and MATA_HMG domains all sharing a common ancestor [26]. The HMGB domain was hypothesized to be the oldest with the SOX and MATA_HMG domain lineages arising later and confined to Metazoa and Fungi, respectively [26]. This places the root of all HMG domains within the HMGB group and allows us to map a direction of time onto the phylogram. MATA_HMG is not a monophyletic group without the inclusion of α1, therefore, because α1 is a subgroup of MATA_HMG we infer that MATA_HMG gave rise to α1.

### Secondary and tertiary structure prediction of the α1 domain suggests it is a HMG domain

**Figure 4:**
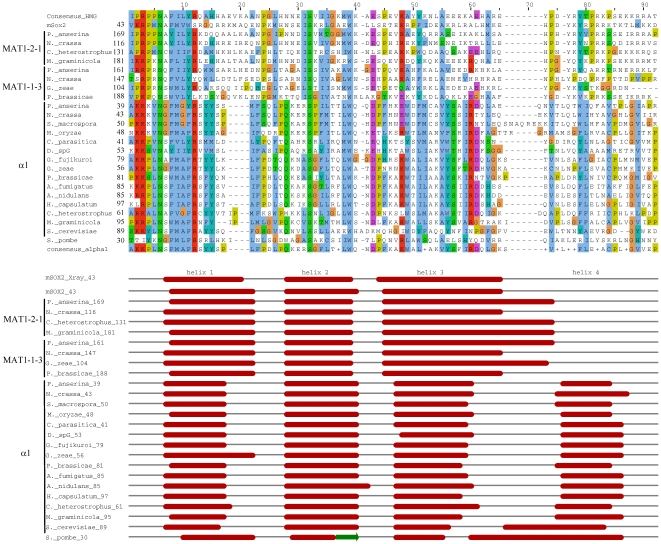
Secondary structure of MATA_HMG and α1 domains from proteins of representative species of Pezizomycotina. The alignment was obtained with ClustalW2 [63] and coloured according to the Clustal X colour scheme provided by Jalview [64]. This colour scheme is displayed in Table S3. The prediction of secondary structures was performed with Jpred3 [27]. All diplayed helices have a JNETCONF score of at least 7, except for helix 2 from *S*. *pombe* which has a JNETCONF score of 0 for all helix 2 positions. The secondary structure presented in the mSOX2_Xray line is from [28] and served to validate accuracy of Jpred3. Full species names and accession numbers are in Table S4.

Sequence conservation between the α1 and HMG domains suggests that they may have similar secondary and tertiary structure. We first examined secondary structure predictions for the MATA_HMG domains from MAT1-2-1 and MAT1-1-3 mating-type proteins with Jpred3 [27]. The three alpha helices that characterize HMG domains [28], [29], [30] were predicted (Figure 4). We then analyzed secondary structures of α1 domains. All α1 domains tested displayed three alpha helices that coincide in position with those obtained with Sox2 (Figure 4), but α1 domains are characterized by a shorter helix 1 and 3, and a fourth alpha helix at the C-terminus. The α1 domain of the *S*. *cerevisiae* Matα1p also displayed these four alpha helices, in agreement with previous secondary structure prediction [14]. The putative α1 domain of *S. pombe* Pc also contained the four helices, however the second has no confidence support (see Figure 4).

Next, the proteins used for secondary structure prediction were submitted to Phyre for fold recognition [31]. As expected, the best matching templates for pezizomycotinan MATA_HMG mating-type proteins (MAT1-2-1 and MAT1-1-3, see Figure 1) were known HMG template structures (Table 1). The α1 proteins also had best matching templates in HMG protein structures (Table 1). Likelihood of the homology is good (95%) and all tested α1 domains had the HMG family fold descriptor. Moreover, for all α1 proteins indicated in Table 1, the top ten highest scoring matches were to known HMG structures (see Table S2 for *P. anserina* FMR1, *N. crassa* mat A-1 and *C. heterostrophus* MAT1-1-1). These results strongly suggest that α1 has HMG structure. Although *S. pombe* Pc protein is classified as an HMG protein in Swissprot (P10841) and our phylogenetic analysis placed it closest to Dothideomycete MATA_HMG, the Pc protein has no significant support as an HMG domain (Table 1). We conclude that classification of Pc as an α1 or HMG protein *sensu stricto* is uncertain, although a relationship to HMG (and therefore to α1) is suggested by the phylogram (Figure 3). Additional examples from taphrinomycotinan species are needed to determine if they encode a new class of HMG-box genes.

**Table 1:** Structure prediction with Phyre of HMG and α1 domains from representative species from major groups of Ascomycota.

Query name (domain)	Fungus^a^	Template^b^ (identity)	E-value ^c^	Estimated precision	Fold/PDBdescriptor
FPR1 (HMG)	*P. anserina*	d2lefa (24%)	2.8e^−14^	100%	HMG
mat a-1 (HMG)	*N. crassa*	d2lefa (18%)	1.5e^−14^	100%	HMG
MAT1-2-1(HMG)	*C. heterostrophus*	d2lefa (19%)	5.6e^−14^	100%	HMG
MAT1-2-1 (HMG)	*M. graminicola*	d2lefa (30%)	8.7e^−15^	100%	HMG
SMR2 (HMG)	*P. anserina*	d2lefa (25%)	1.1e^−14^	100%	HMG
mat A-3 (HMG)	*N. crassa*	d2lefa (20%)	9.9e^−14^	100%	HMG
MAT1-1-3 (HMG)	*G. zeae*	d2lefa (19%)	1.8e^−13^	100%	HMG
MAT1-1-3/phb1 (HMG)	*P. brassicae*	d2lefa (23%)	4.9e^−15^	100%	HMG
FMR1 (α1)	*P. anserina*	d1qrva (12%)	0.005	95%	HMG
mat A-1 (α1)	*N. crassa*	d1qrva (11%)	0.028	95%	HMG
SMT A-1 (α1)	*S. macrospora*	d1qrva (11%)	0.0043	95%	HMG
MAT1-1-1 (α1)	*M. oryzae*	d1qrva (14%)	0.026	95%	HMG
MAT1-1-1 (α1)	*C. parasitica*	d1qrva (10%)	0.017	95%	HMG
MAT1-1-1 (α1)	*D. sp*	d2gzka2 (14%)	0.0022	95%	HMG
MAT1-1-1 (α1)	*G. fujikuroi*	d1qrva (18%)	0.014	95%	HMG
MAT1-1-1 (α1)	*G. zeae*	d1qrva (15%)	0.0052	95%	HMG
MAT1-1-1/pad1 (α1)	*P. brassicae*	d1qrva (18%)	0.0025	95%	HMG
MAT1-1-1 (α1)	*A. fumigatus*	d1qrva (15%)	0.012	95%	HMG
MAT1-1/MATB (α1)	*A. nidulans*	d1qrva (15%)	0.0016	95%	HMG
MAT1-1-1 (α1)	*H. capsulatum*	d1qrva (14%)	0.014	95%	HMG
MAT1-1-1 (α1)	*C. heterostrophus*	d1qrva (15%)	0.0013	95%	HMG
MAT1-1-1 (α1)	*M. graminicola*	d1qrva (19%)	0.0059	95%	HMG
Matα1p (α1)	*S. cerevisiae*	d1k99a (12%)	0.0086	95%	HMG
Pc (HMG)	*S. pombe*	d2lefa (14%)	4	45%	HMG

a For complete names and accession numbers, see Table S4.

b Highest scoring template to the query. Templates are known structures from the PHYRE fold library; d2lefa, lymphoid enhancer-binding factor, LEF1 from Mouse (*Mus musculus*); d1qrva, HMG-D from *Drosophila melanogaster*; d2gzka2, SRY from Human (*Homo sapiens*); d1k99a, nucleolar transcription factor 1 (Upstream binding factor 1, UBF-1) from Human (*H. sapiens*). The percentage sequence identity between the query and template is displayed in brackets. This is calculated relative to the shortest sequence.

C likelihood of structural homology.

**Figure 5:**
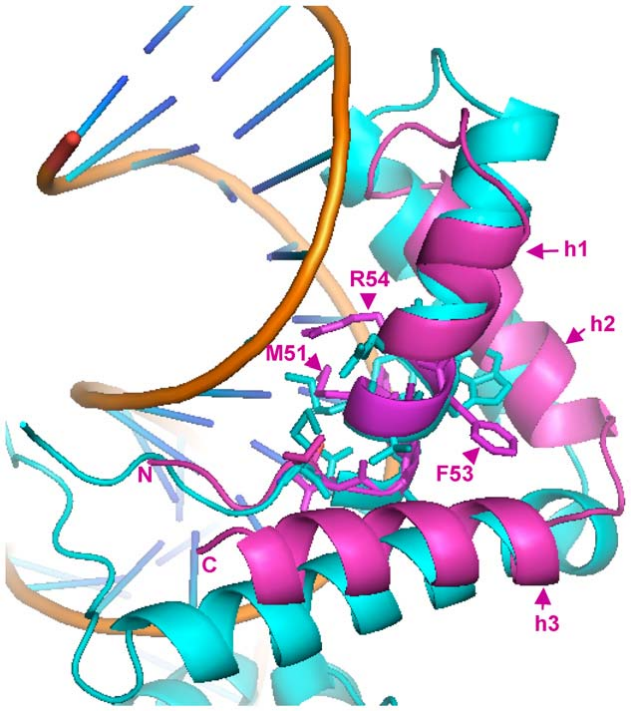
3D-structure of the α1 domain from MAT1-1-1/mat A-1 of *N. crassa*. Schematic ribbon presentation of the superposition of the α1 domain (magenta) onto the structure of the Sox2 HMG domain (cyan) as observed in the tertiary DNA/Sox2/Oct1(POU domain) complex. DNA is represented as gold ribbons (polyphosphate) and blue sticks (bases). Amino acid residues important for DNA recognition and bending are represented as sticks. Residues (methionine M51, phenylalanine F53 and arginine R54) putatively important for function are labelled. Numbering is from the N-terminus methionine. Alpha helices are labelled h1, h2 and h3. Accession number: AAC37478, 3D structure established from residue 44 to 97.

To further search for structural homologs of the α1 domain we submitted the *N. crassa* α1 protein (mat A-1) sequence to the I-Tasser Structure Prediction Meta Server [32]. All best scoring templates for the α1 domain were structures of HMG proteins. When we iterated this search using Rosetta [33] and FUGUE [34], both predicted that the α1 domain has an HMG-like architecture (data not shown). In Figure 5 we show a model of the mat A-1 α1 domain superimposed upon the HMG domain of the transcription factor Sox2 in a ternary complex with an oligonucleotide and the POU DNA-binding domain of the OCT1 transcription factor [35]. HMG-box proteins have an L-shaped fold, comprising three alpha helices, stabilized by a hydrophobic core. Helix 3 and the N-terminal strand form the long arm of the L, while the short arm of the L is formed by helices 1 and 2. Helices 2 and 3 are approximately orthogonal to each other. Non-structured peptide extensions are usually present at the N- and C-terminal ends. These peptides become ordered upon DNA binding and occupy minor and major grooves. The first two helices are about the same length but the third one is much longer. Helix one is bent. Various structures of HMG-domain DNA complexes have shown that the structure of the HMG-core is maintained upon DNA binding.

The α1 domain 3D model, as proposed by the I-Tasser prediction server, has some notable differences with the canonical HMG-domain fold. The first alpha helix of the α1 domain is shorter by about one helical turn compared to its counterpart in HMG-domain proteins and the third helix is about half as long as the corresponding helix in canonical HMG domains (Figure 5). In total, the α1 domain sequences are shorter by about 30 residues than those of the canonical HMG domain and thus may therefore be described as truncated HMG domains. It is unknown whether α1 domains directly contact DNA, but from the model it can be predicted that the α1-domain should be able to bind DNA in a manner similar to canonical HMG domains. In support of this, we note that the DNA-binding core motifs for the *N. crassa* MATA_HMG mat a-1 and *S. cerevisiae* Matα1p are CAAAG [36] and CAATG [12], respectively.

3D-structures for a number of mammalian HMG-DNA complexes have been determined, including Sox2 [28] used in Figure 4, HMG-D [37], LEF-1 [38] and SRY [30]. In all cases, the HMG domain binds to the minor groove of DNA and introduces severe bends toward the major groove. Side chains from residues of helix 1 and helix 2 are inserted between base-pair stacks of the recognition sequence. However, the C-terminal region of each of these proteins interacts differently with its DNA target. For instance, for HMG-D, which binds DNA without sequence specificity, the C-terminal helix does not interact, while for LEF-1 it lies in the compressed major groove and stabilizes the bent conformation. Sequence specific HMG domains intercalate a hydrophobic residue between two bases of the (A/T)(A/T)CAAAG [39] recognition sequence. These residues are either Met, Ile or Val (position 9 in Figures 2A and 4) and these are flanked by aromatic residues at positions −1 and +2. These aromatics firmly anchor the recognition helix into the hydrophobic core. Remarkably, the aromatic residues at positions -1 and +2 from the conserved position 9 (Met in mat A-1) are present in the first helical turn of the α1 domain of mat A-1 (Figure 5) and a derived consensus is highly conserved in all α1 sequences (F-[MIV]-[AG]-F, Figures 2A and 4). Superimposition of the α1-domain model of *N. crassa* onto the structure of the Sox2-DNA complex (Figure 5) shows the Met (M51) and Phe (F53) could play the same role in DNA bending as the corresponding amino acids in conventional HMG boxes. Alignment of HMG and α1 sequences reveals a highly conserved Arg (position 12 in Figures 2A and 4, R54 in Figure 5). This Arg contacts the DNA phosphate backbone in all documented HMG-DNA structures. As shown in Figure 5 its position in the model of the α1 domain suggests a similar functional role. Additional data confirming the similar structure of α1 and HMG domains are presented in Figure S2. *Fusarium sacchari* α1 and *Aspergillus flavus* MATA_HMG domains were used as representative candidates for structure prediction. Superimposition of their structure showed considerable overlap (C in Figure S2). The α1 domain overlaps also the SOX17 structure (D in Figure S2). Thus, secondary and tertiary structural analyses support the conclusion, reached using phylogenetic approaches, that α1 domain proteins belong to the HMG family of proteins. We propose to incorporate these proteins in a new subclass of HMG proteins termed MATα_HMG.

### MAT1-1-1 proteins contain a second conserved region in addition to the α1 domain

**Figure 6:**
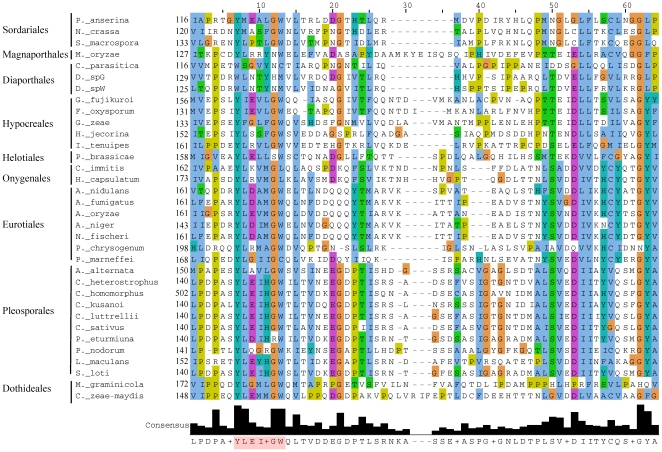
Alignment of the conserved region downstream of the MATα_HMG region of representative species from major groups of Pezizomycotina. The alignment was obtained with ClustalW2 [63] and coloured according to the Clustal X colour scheme provided by Jalview [64]. This colour scheme is displayed in Table S3. The Y-[LMIF]-x3-G-[WL] motif is highlighted in pink in the consensus line. Accession numbers for MAT1-1-1 proteins: *Podospora anserina* (CAA45519), *Neurospora crassa* (AAC37478), *Sordaria macrospora* (CAA71623), *Magnaporthe oryzae* (strain 70-6) (BAC65087), *Cryphonectria parasitica* (AAK83346), *Diaporthe spG* (BAE93756), *Diaporthe spW* (BAE93750), *Gibberella fujikuroi* (AAC71055), *Fusarium oxysporum* (BAA75910), *Gibberella zeae* (AAG42809), *Hypocrea jecorina* (ACR78244), *Isaria tenuipes* (BAC67541), *Pyrenopeziza brassicae* (CAA06844), *Coccidioides immitis* (ABS19618), *Histoplasma capsulatum* (AB087596), *Aspergillus nidulans* (EAA63189), *Aspergillus fumigatus* (AAX83122), *Aspergillus oryzae* (Q2U537), *Aspergillus niger* (XP_001394976), *Neosartorya fischeri* (ABQ28692), *Penicillium chrysogenum* (CAP17332), *Penicillium marneffei* (ABC68484), *Alternaria alternata* (BAA75907), *Cochliobolus heterostrophus* (CAA48465), *Cochliobolus homomorphus* (AAD33441), *Cochliobolus kusanoi* (AAD33443), *Cochliobolus luttrellii* (AAD33439), *Cochliobolus sativus* (AAF87723), *Pleospora eturmiuna* (AAR00973), *Phaeosphaeria nodorum* (AAO31740), *Leptosphaeria maculans* (AAO37757), *Stemphylium loti* (AAR04470), *Mycosphaerella graminicola* (AAL30838), *Cercospora zaea-maydis* (ABB83705).

The alignment of the MATα_HMG proteins reveals a conserved region spanning approximately 60 residues, immediately adjacent to and downstream of the fourth alpha helix of the MATα_HMG domain in pezizomycotinan proteins (Figure 4). The region consists of a core conserved motif Y-[LMIF]-x(3)-G-[WL], and less conserved residues covering a larger region (Figure 6). *S*. *cerevisiae*, *Pichia angusta* and *Candida albicans* MATα_HMG proteins stop 7, 14 and 15 residues, respectively, after the end of the MATα_HMG domain and therefore do not include this 60 residue conserved region. Alignment of the 59 and 88 residues downstream of MATα_HMG domain from *Kluyveromyces lactis* and *Yarrowia lipolytica*, respectively, failed to reveal the conserved region in these species (data not shown). Moreover, ScanProsite [40] did not detect the Y-[LMIF]-x(3)-G-[WL] motif in MATα_HMG proteins of *S. cerevisiae, P. angusta, C. albicans, K. lactis* or *Y. lipolytica.* Taken together, these observations support the idea that this conserved region is specific to Pezizomycotina. Analysis of currently available MATα_HMG proteins from Diaporthales indicates that the core consensus Y-[LMIF]-x(3)-G-[WL] is either modified or lost in this group, although the larger conserved region is present (Figure 6). Screening of entire *Diaporthe sp*. MATα_HMG proteins [41] with ScanProsite failed to detect the core consensus motif. A similar search performed on *C*. *parasitica* protein [42] revealed the motif Y-L-N-L-A-G-T starting at position 106. Additional examples from diaporthale mating types are needed to determine a possible new core consensus motif. Conservation of this region was noted previously (and designated as HMGB) by Turgeon and Lu and reported in [43], [44]. These authors hypothesized that it resembles an HMG domain. Prediction of HMGB secondary structures with Jpred3 [27] and modelling with the I-Tasser Structure Prediction Meta Server [32], however, does not reveal the characteristic secondary and tertiary structures of HMG domains (data not shown). Further analyses will be necessary to establish the structure and origin of this region. Data obtained from mutations in the MATα_HMG-box gene of *N. crassa* (*mat A-1*) suggest that this conserved region is necessary for male, but not female, fertility [45]. For the MATα_HMG protein of *C. heterostrophus*, changing the conserved tryptophan (W) residue to alanine or arginine in the Y-[LM]-x(3)-G-[WL] core motif affects the number and development of pseudothecia, supporting the importance of this region for protein function (unpublished, Liu and Turgeon).

### Mating-type evolution in the fungal kingdom

**Figure 7:**
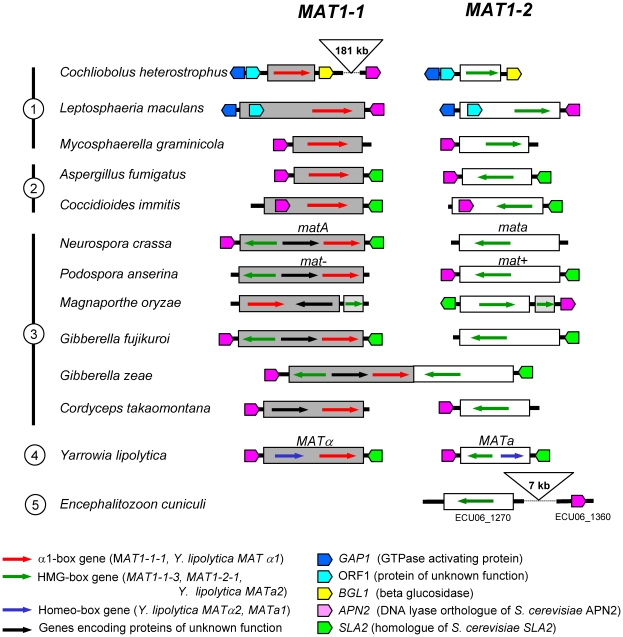
Mating-type loci and DNA lyase gene position in representative species of Ascomycota. The DNA lyase orthologs are indicated only when confirmed by sequencing. The physical linkage of the *MAT* locus and the DNA lyase gene may be relaxed, as exemplified by *Cochliobolus heterostrophus*, where the two genes are separated by 181 kb. Orthology of DNA lyase genes was determined by FUNGIpath [52]. Mating-type structures were compiled for the following species and corresponding references: *C. heterostrophus* [68], *Leptosphaeria maculans* [69], *Mycosphaerella graminicola* [70], *Aspergillus fumigatus* [71], *Coccidioides immitis* [72], *Neurospora crassa* [49], [50], *Podospora anserina* [43], *Magnaporthe oryzae* [43], *Gibberella fujikuroi* [73], *Gibberella zeae* [50], *Cordyceps takaomontana* [74], *Yarrowia lipolytica* [49], *Encephalitozoon cuniculi* [46]. Circled figures on the left: 1: Dothideomycetes; 2: Eurotiomycetes; 3: Sordariomycetes; 4: Saccharomycetales; 5: Microsporidia. Linkage of *C. heterostrophus MAT1-1-1* to *DNA lyase* gene (ESTEXT_GENEWISE1PLUS.C_40361) was determined from the sequence data produced by the US Department of Energy Joint Genome Institute http://www.jgi.doe.gov/. Linkage of *G. fujikuroi (Fusarium verticillioides) MAT1-1-1* to *DNA lyase* gene (FVEG_02488) was determined from the version 1 sequence data produced by the Broad Institute http://www.broadinstitute.org/annotation/genome/fusarium_group/MultiHome.html.

Idnurm and co-workers proposed that HMG domain proteins might represent the ancestral fungal sex determinant based on the discovery of HMG-box genes at the *MAT* locus in early diverged branches of fungi [17], [46]. This model and subsequent analyses [47], [48], however, do not explain the acquisition of α1-box genes in ascomycotan mating types. Low similarities between α1 and HMG domains have been noticed previously and a relationship suggested [3], [4], [17], although this contention has not been carefully examined. Sequence and phylogenetic analyses and structural modelling presented here substantiate the hypothesis that the evolutionary origin of α1 is in the HMG domain, thus providing a clue to the origin of the α1-box genes. This hypothesis is in agreement with the model proposed by Idnurm and co-workers [17]. However this model is strengthened by data which reveal linkage conservation of certain genes flanking the mating-type locus in Microsporidia and Ascomycota. A gene encoding a DNA lyase is immediately adjacent to *MAT* of many Ascomycota [43], [49], [50] (Figure 7). Remarkably, the analysis of the environment of the putative mating-type locus of *Encephalitozoon cuniculi* (Microsporidia) reveals the presence of an homolog of the DNA lyase encoding genes [46]. This gene is 7 kb away from the *E. cuniculi* putative *MAT* locus [51] (Figure 7) and analysis with FUNGIpath [52] confirmed that it is an ortholog of the DNA lyases genes adjacent to *MAT* loci in Ascomycotina. Although synteny *sensu stricto* is not conserved between Microsporidia and Ascomycota mating types, the presence of these orthologous DNA lyase encoding genes in the vicinity of the mating-type locus in Microsporidia and Ascomycota is highly significant and strongly supports a common origin.

### Conclusion

The model proposed by Lee et al. [53] for early steps of mating type formation should result in idiomorphic or allelic sequences of a given mating-type locus containing phylogenetically related genes. The presence of MATα_HMG and MATA_HMG-box genes in ascomycotan opposite mating types (Figure 1) is in agreement with this model. Only a few mating types are an exception to this rule; ironically, the most prominent example is S. cerevisiae MAT, one of the most thoroughly characterized loci in terms of MAT regulation. It lacks the MATα2 (MATA_HMG-box) gene [49] (Figure 1), but has evolved alternative transcriptional circuits ensuring appropriate mating-type target gene expression [54].

The identification of the MATα_HMG structure is an additional example of a study confirming that protein spatial structure is more conserved than amino acid sequences (reviewed in [55]), as suggested first by Lesk and Chothia [56]. Functional conservation acts as a strong restraint limiting sequence and, even more, structural divergence [57]. It must be noted, however, that there are some differences between the predicted MATα_HMG structure and SOX2 folding, in particular the presence of a fourth alpha helix. Experimental determination of crystal structure of the MATα_HMG domain is in progress and should help in understanding the function of this additional helix. It is surprising that the MATA and MATα_HMG sequences are so much divergent, especially when paralogous MATA and MATα_HMG proteins encoded by opposite idiomorphs are considered. It is worth noting that the term of idiomorph was indeed proposed by Metzenberg and Glass in 1990 to denote that mating-type sequences “are not obviously related by structure or common descent” [1]. Further investigations will be necessary to identify the factors that favored MATα_HMG divergence and have thwarted the determination of its origin for such a long time.

## Materials and Methods

### Sequence acquisition

Initially, we retrieved and aligned ∼200 residues from five α1 and ∼75 residues from five MATA_HMG domains, from selected Ascomycetes (Figure S1). Alignment with Kalign [58] revealed a core region of ∼40 amino acids with conserved signatures starting at position 1-2 and 11-48 in the MATA_HMG and α1 sequences, respectively (Figure S1). Sequences annotated as α1 (MAT_Alpha1) or HMG (MATA_HMG, SOX-TCF_HMG, or HMGB-UBF_HMG) in the NCBI database were collected. The core region of ∼40 amino acids was aligned for all sequence sets using Muscle [18]; sequences with less than 80% coverage of the core were removed. HMGB-UBF HMG-domain sequences contained a small section of varying size within the core region that was removed to create a compact alignment with conserved sections only. The resulting core region dataset consisted of 300 α1 (Dataset S1), 257 MATA_HMG (Dataset S2), 3,054 SOX_HMG (Dataset S3) and 2,162 HMGB_HMG sequences (Dataset S4).

### Identifying consensus amino acids

Conserved amino acids were estimated with WebLogo [19] using core region data sets. The resultant logos were taken as the consensus sequence for each of the domains. The α1 domain consensus was divided into two; one corresponded to α1 in the Pleosporales and the second to α1 in all other Pezizomycotina. COMPASS was used for profile-profile analysis [21].

### E-value computing

Alignments were performed using the NCBI BLASTP suite-2 tool [59].

### Ancestral sequence prediction

Input for this were sequences corresponding to ascomycete α1 and MATA_HMG domains. The datasets contained domains from Sordariomycetes, Leotiomycetes, Eurotiomycetes, Dothideomycetes, Pezizomycetes, Saccharomycotina and Taphrinomycotina and represented a broad range of species. Sequences were input as independent HMG and α1 datasets. The predicted ancestral amino acid sequences of the ascomycete α1 and HMG domains were determined using the Ancescon ancestral protein predictor [22]. Statistical alignments were performed using the NCBI BLASTP suite-2 sequences [59].

### Phylogenetic analysis

Randomly selected and certain selected core sequences from the α1 and HMG core region datasets were aligned using Kalign [58]. ProtTest v2.4 identified LG+G and LG+I+G as the best models for the data [23]. Trees were produced using both models with TREEFINDER using maximum likelihood, selected models and 10,000 replicates producing concurrent trees with the LG+G tree shown [60]. Phylograms were viewed using TreeView 1.6.6 [61]. Local rearrangement of expected likelihood weights (LR-ELW) edge support were used as confidence in configuration of branches [62]. Alternative topologies were tested using the KH and SH tests in TREEFINDER [24], [25].

### Structure prediction

Sequence alignments were obtained with ClustalW2 [63], colours with Jalview [64] and structure prediction with Jpred3 [27]. These tools were provided by EBI on http://www.ebi.ac.uk/services/. Fold recognitions, 3D structure predictions and motif searches were performed with Phyre [31], I-Tasser Structure Prediction Meta Server [32] and ScanProsite [40], respectively.

### Orthologous gene analysis

The orthology of DNA lyase proteins was determined with FUNGIpath [52].

## Supporting Information

Figure S1 **Initial alignment of MATA_HMG and α1 domains used to identify a conserved core region**. ClustalW2 [63] alignment of complete α1 and HMG domains from five α1 and five MATA_HMG sequences. Identical amino acids across all sequences are coloured blue, >5 identical or similar amino acids are coloured grey. Core region indicated with *. Accession numbers for MATA_HMG: *Pyrenopeziza brassicae* MAT1-2-1/phb2 (CAA06843), *Neurospora crassa* MAT1-2-1/mat a-1 (AAA33598), *Mycosphaerella graminicola* MAT1-2-1 (AAL30836), *Podospora anserina* MAT1-1-3/SMR2 (CAA52051), *Cochliobolus heterostrophus* MAT1-2-1 (CAA48464). Accession numbers for α1: *Podospora anserina* FMR1 (CAA45519), *N. crassa* mat A-1 (AAC37478), *Alternaria alternata* (O94160), *Cochliobolus ellisii* (Q9Y8C7), *Fusarium oxysporum* (O59851).
(TIF)

Figure S2 **Tertiary structure predictions of α1 and MATA_HMG domains.** Images were made using PyMOL [75]. Amino acids of the conserved signature motif identified in Figure 1B are highlighted in yellow. N and C terminal ends are labeled. (A) PHYRE [31] structure prediction for *Fusarium sacchari* α1 domain (accession number: 97974007, residues 35 to 235). (B) PHYRE [31] structure prediction for *Aspergillus flavus* MATA-HMG domain (accession number: XP_002374195, residues 141 to 200). (C) Superimposition of structures from A and B showing considerable overlap. The first alpha1 helix is shorter than the equivalent in MATA-HMG. (D) Crystallized structure of mouse SOX17 in green in direct contact with DNA in orange [76].
(TIF)

Table S1 **Accession numbers for proteins of** **Figure 3** **.**
(DOC)

Table S2 **Top ten scoring with PHYRE for selected α1 domains.**
(DOC)

Table S3 **Color scheme used for Jalview.**
(DOC)

Table S4 **Accession numbers for proteins of** **Figure 4** **,** **Table 1** **and Table S2.**
(DOC)

Dataset S1 **α1 sequences used for α1 core region determination.**
(XLS)

Dataset S2 **MATA_HMG sequences used for HMG core region determination.**
(XLS)

Dataset S3 **SOX_HMG sequences used for HMG core region determination.**
(XLS)

Dataset S4 **HMGB_HMG sequences used for HMG core region determination.**
(XLS)
