# Supplementary figures and images for: Tracing the Origin of the Fungal α1 Domain Places Its Ancestor in the HMG-Box Superfamily: Implication for Fungal Mating-Type Evolution
Source: PLoS One. 2010 Dec 8;5(12):e15199. doi: 10.1371/journal.pone.0015199 (PMC2999568; doi:10.1371/journal.pone.0015199)

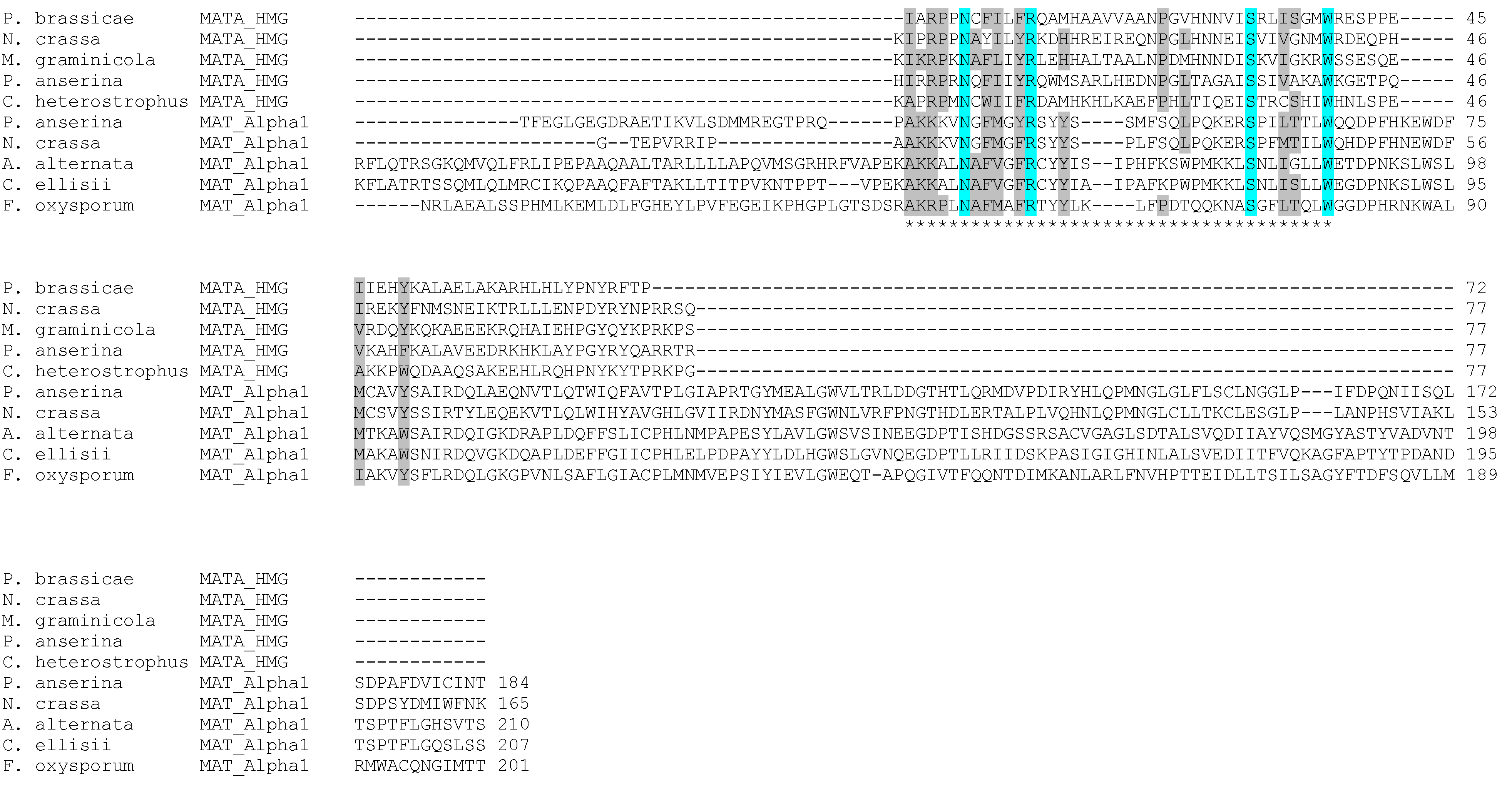

Supplement: Figure S1 — Initial alignment of MATA_HMG and α1 domains used to identify a conserved core region. ClustalW2 [63] alignment of complete α1 and HMG domains from five α1 and five MATA_HMG sequences. Identical amino acids across all sequences are coloured blue, >5 identical or similar amino acids are coloured grey. Core region indicated with *. Accession numbers for MATA_HMG: Pyrenopeziza brassicae MAT1-2-1/phb2 (CAA06843), Neurospora crassa MAT1-2-1/mat a-1 (AAA33598), Mycosphaerella graminicola MAT1-2-1 (AAL30836), Podospora anserina MAT1-1-3/SMR2 (CAA52051), Cochliobolus heterostrophus MAT1-2-1 (CAA48464). Accession numbers for α1: Podospora anserina FMR1 (CAA45519), N. crassa mat A-1 (AAC37478), Alternaria alternata (O94160), Cochliobolus ellisii (Q9Y8C7), Fusarium oxysporum (O59851). (TIF) [file pone.0015199.s001.tif]

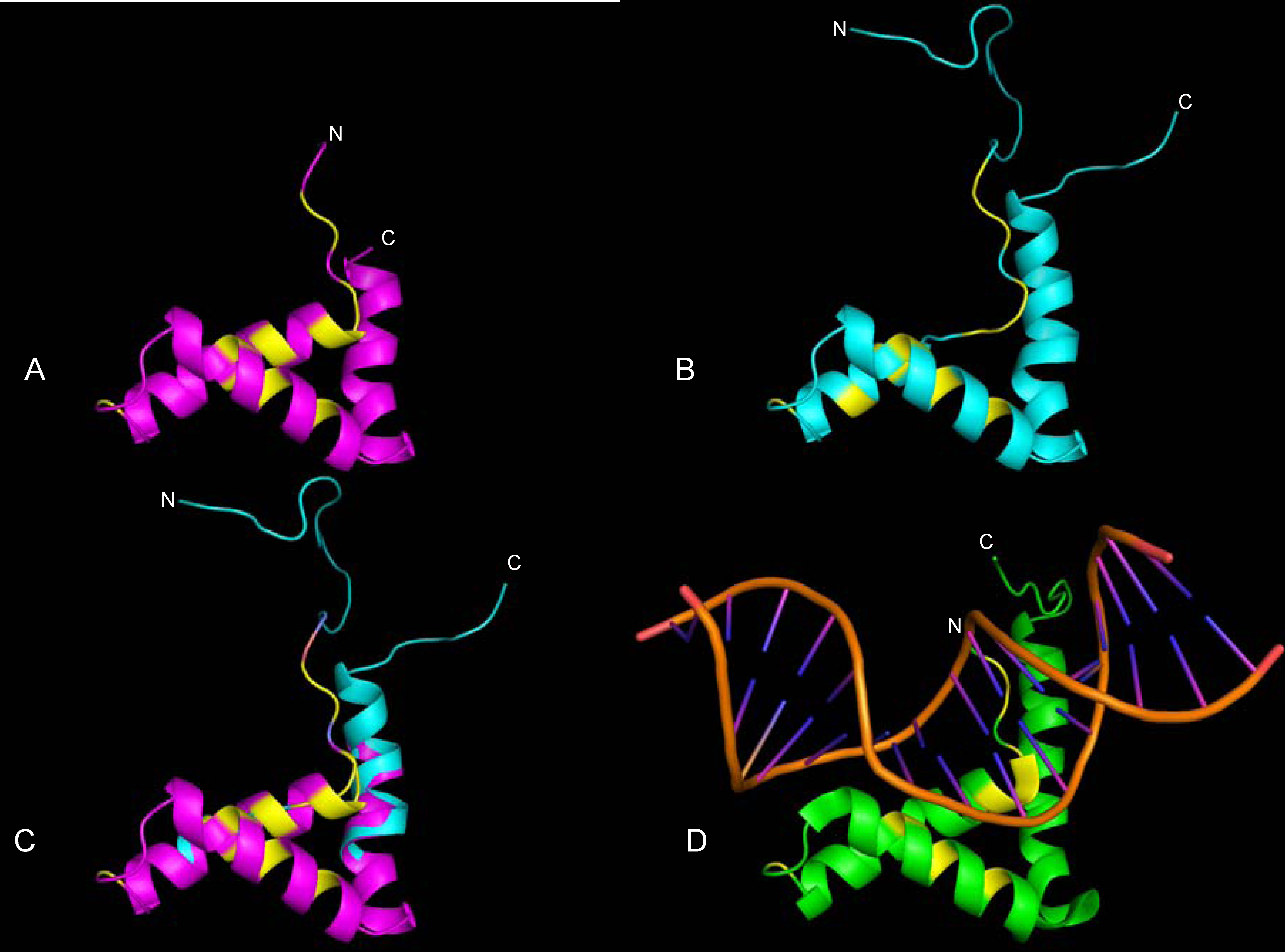

Supplement: Figure S2 — Tertiary structure predictions of α1 and MATA_HMG domains. Images were made using PyMOL [75]. Amino acids of the conserved signature motif identified in Figure 1B are highlighted in yellow. N and C terminal ends are labeled. (A) PHYRE [31] structure prediction for Fusarium sacchari α1 domain (accession number: 97974007, residues 35 to 235). (B) PHYRE [31] structure prediction for Aspergillus flavus MATA-HMG domain (accession number: XP_002374195, residues 141 to 200). (C) Superimposition of structures from A and B showing considerable overlap. The first alpha1 helix is shorter than the equivalent in MATA-HMG. (D) Crystallized structure of mouse SOX17 in green in direct contact with DNA in orange [76]. (TIF) [file pone.0015199.s002.tif]
