# Supplementary material for: Tracing the Origin of the Fungal α1 Domain Places Its Ancestor in the HMG-Box Superfamily: Implication for Fungal Mating-Type Evolution
Source: PLoS One. 2010 Dec 8;5(12):e15199. doi: 10.1371/journal.pone.0015199 (PMC2999568; doi:10.1371/journal.pone.0015199)
Supplement: Table S1 — Accession numbers for proteins of Figure 3 . (DOC) [file pone.0015199.s003.doc]

| Group | Code | Protein name | Domain | Organism | Accession number |
| --- | --- | --- | --- | --- | --- |
| Sordariales | Neucr1 | MAT1-1-1/mat A-1 | α1 | *Neurospora crassa* | AAC37478 |
|  | Neucr2 | MAT1-2-1/mat a-1 | MATA_HMG | *Neurospora crassa* | AAA33598 |
|  | Neucr3 | MAT1-1-3/mat A-3 | MATA_HMG | *Neurospora crassa* | AAC37476 |
|  | Podan1 | MAT1-1-1/FMR1 | α1 | *Podospora anserina* | CAA45519 |
|  | Podan2 | MAT1-2-1/FPR1 | MATA_HMG | *Podospora anserina* | CAA45520.1 |
|  | Podan3 | MAT1-1-3/SMR2 | MATA_HMG | *Podospora anserina* | CAA52051 |
|  | Sorma1 | SMTA-1 | α1 | *Sordaria macrospora* | CAA71623 |
|  | Sorma2 | SMTa-1 | MATA_HMG | *Sordaria macrospora* | CAA71624.1 |
| Magnaporthales | Magor1 | MAT1-1-1 | α1 | *Magnaporthe oryzae* | BAC65083.1 |
|  | Magor2 | MAT1-2-1 | MATA_HMG | *Magnaporthe oryzae* | BAC65094.1 |
| Diaporthales | Crypa1 | MAT1-1-1 | α1 | *Cryphonectria parasitica* | AAK83346 |
|  | Crypa3 | MAT1-1-3 | MATA_HMG | *Cryphonectria parasitica* | AAK83344.1 |
| Hypocreales | Fusac3 | MAT1-1-3 | MATA_HMG | *Fusarium acaciae-mearnsii* | ABE98373.1 |
|  | Gibfu1 | MAT1-1-1 | α1 | *Gibberella fujikuroi* | AAC71055 |
|  | Gibfu3 | MAT1-1-3 | MATA_HMG | *Gibberella fujikuroi* | AAC71053.1 |
|  | Gibze1 | MAT1-1-1 | α1 | *Gibberella zeae* | AAG42809 |
|  | Gibze3 | MAT1-1-3 | MATA_HMG | *Gibberella zeae* | AAG42812 |
| incertae sedis | Verda2 | MAT1-2-1 | MATA_HMG | *Verticillium dahliae* | BAG12301.1 |
| Helotiales | Botfu5 | HMG | HMGB | *Botryotinia fuckeliana* | XP_001548220 |
|  | Pyrbr1 | MAT1-1-1/pad1 | α1 | *Pyrenopeziza brassicae* | CAA06844 |
|  | Pyrbr3 | MAT1-1-3/phb1 | MATA_HMG | *Pyrenopeziza brassicae* | CAA06846.1 |
|  | Rhyse1 | MAT1-1-1 | α1 | *Rhynchosporium secalis* | CAD71141.1 |
| Eurotiales | Aspfu1 | MAT1-1-1 | α1 | *Aspergillus fumigatus* | AAX83123.1 |
|  | Aspfu2 |  | MATA_HMG | *Aspergillus fumigatus* | XP_751745.1 |
|  | Aspni1 | MAT1-1/MATB | α1 | *Aspergillus nidulans* | EAA63189.1 |
|  | Aspni2 | MAT1-2/MATA | MATA_HMG | *Aspergillus nidulans* | CBF85903.1 |
|  | Penma1 | MAT1-1-1/MAT-1 | α1 | *Penicillium marneffei* | ABC68484.1 |
|  | Penma2 |  | MATA_HMG | *Penicillium marneffei* | XP_002151220.1 |
| Onygenales | Ajeca1 | MAT1-1-1 | α1 | *Ajellomyces capsulatus* | ABO87596.1 |
|  | Ajeca2 | MAT1-2-1 | MATA_HMG | *Ajellomyces capsulatus* | EER39720.1 |
| Pleosporales | Altal1 | MAT1-1-1 | α1 | *Alternaria alternata* | BAA75907.1 |
|  | Altal2 | MAT1-2-1 | MATA_HMG | *Alternaria alternata* | BAA75903.1 |
|  | Altbr1 | MAT1-1-1 | α1 | *Alternaria brassicicola* | AAK85542.1 |
|  | Bipsa2 | MAT1-2-1 | MATA_HMG | *Bipolaris sacchari* | CAA65081.1 |
|  | Coche1 | MAT1-1-1 | α1 | *Cochliobolus heterostrophus* | CAA48465 |
|  | Coche2 | MAT1-2-1 | MATA_HMG | *Cochliobolus heterostrophus* | CAA48464.1 |
|  | Cocho2 | MAT1-2/1 | MATA_HMG | *Cochliobolus homomorphus* | AAD33441.1 |
|  | Pyrte2 | MAT1-2-1 | MATA_HMG | *Pyrenophora teres* | AAY35017 |
|  | Stesa1 | MAT1-1 | α1 | *Stemphylium sarciniforme* | AAR04460 |
| Dothideales | Dotpi2 | MAT1-2-1 | MATA_HMG | *Dothistroma pini* | ABK91353 |
|  | Mycgr1 | MAT1-1-1 | α1 | *Mycosphaerella graminicola* | AAL30838 |
|  | Mycgr2 | MAT1-2-1 | MATA_HMG | *Mycosphaerella graminicola* | AAL30836.1 |
| Saccharomycotina | Canal1 | MTL α1 | α1 | *Candida albicans* | XP_714749 |
|  | Canal2 | Rfg1p | MATA_HMG | *Candida albicans* | XP_715804.1 |
|  | Lacth1 | MATα1 | α1 | *Lachancea thermotolerans* | XP_002554225.1 |
|  | Sacce1 | Matα1p | α1 | *Saccharomyces cerevisiae* | EDN62161.1 |
|  | Sacce2 | Rox1p | MATA_HMG | *Saccharomyces cerevisiae* | NP_015390.1 |
|  | Sacce5a | NHP6A | HMGB | *Saccharomyces cerevisiae* | EDN61184 |
|  | Sacce5b | NHP6B | HMGB | *Saccharomyces cerevisiae* | NP_010459 |
|  | Zygro1 | MAT α1 | α1 | *Zygosaccharomyces rouxii* | XP_002497889.1 |
| Taphrinomycotina | Pneca2 | STE11 | MATA_HMG | *Pneumocystis carinii* | Q870J1 |
|  | Schja2 | STE11 | MATA_HMG | *Schizosaccharomyces japonicus* | XP_002175130 |
|  | Schpo2 | STE11 | MATA_HMG | *Schizosaccharomyces pombe* | CAA77507.1 |
|  | Schpo6 | Pc | HMG | *Schizosaccharomyces pombe* | P10841 |
| Basidiomycota | Ustma2 | Prf1 | MATA_HMG | *Ustilago maydis* | AAC32736 |
| Zygomycota | Phybl8 | SexM | HMG | *Phycomyces blakesleeanus* | ABX27909.1 |
|  | Phybl9 | SexP | HMG | *Phycomyces blakesleeanus* | ABX27912.1 |
| Microsporidia | Antlo7 | HMG | HMG | *Antonospora locustae* | ACI87876.1 |
|  | Entbi7 | Sex locus | HMG | *Enterocytozoon bieneusi* | ACI87872 |
|  | Enccu7 | Sex locus | HMG | *Encephalitozoon cuniculi* | NP_585883 |
| Animalia | Ailme4 | hypothetical | SOX | *Ailuropoda melanoleuca* | EFB23328 |
|  | Anoga4 | AGAP003896-PA | SOX | *Anopheles gambiae* | XP_001230616.1 |
|  | Caeel5 | HMG-4 | HMGB | *Caenorhabditis elegans* | NP_498633 |
|  | Cerel4 | SRY | SOX | *Cervus elaphus yarkandensis* | ABK91721 |
|  | Ciosa4 | TF | SOX | *Ciona savignyi* | NP_001071831.1 |
|  | Culqu4 | pangolin | SOX | *Culex quinquefasciatus* | XP_001864781 |
|  | Danre4 | TF-7 | SOX | *Danio rerio* | AAI63927 |
|  | Danre5a | RNA Polymerase 1 | HMGB | *Danio rerio* | CAQ14015 |
|  | Danre5b | TF | HMGB | *Danio rerio* | NP_957297 |
|  | Danre5c | TF | HMGB | *Danio rerio* | NP_957297.1 |
|  | Drome4a | pangolin | SOX | *Drosophila melanogaster* | NP_001014685.1 |
|  | Drome4b | bobby sox | SOX | *Drosophila melanogaster* | NP_001027087.1 |
|  | Homsa5a | RNA Polymerase 1 | HMGB | *Homo sapiens* | EAW51616 |
|  | Homsa5b | TF1 | HMGB | *Homo sapiens* | NP_055048.1 |
|  | Homsa5c | HMG | HMGB | *Homo sapiens* | 2CS1_A |
|  | Musmu4a | lymphoid enhancer | SOX | *Mus musculus* | EDL12207.1 |
|  | Musmu4b | SOX-1 | SOX | *Mus musculus* | NP_033259.2 |
|  | Musmu4c | HMG | SOX | *Mus musculus* | NP_694878.2 |
|  | Strpu4 | Tcf/Lef | SOX | *Strongylocentrotus purpuratus* | NP_999640.1 |
|  | Takru4 | SOX8b | SOX | *Takifugu rubripes* | AAQ18506 |
|  | Xenla4 | XTCF-3b | SOX | *Xenopus laevis* | CAA67689 |
|  | Xenla5a | TF1-B | HMGB | *Xenopus laevis* | NP_001079429 |
|  | Xenla5b | ubtf-b | HMGB | *Xenopus laevis* | AAH42232 |
|  | Xenla5c | ubtf-b | HMGB | *Xenopus laevis* | AAH42232.1 |
| Planta | Arath5 | HMG | HMGB | *Arabidopsis thaliana* | AAK43965.1 |
