# Supplementary material for: Tracing the Origin of the Fungal α1 Domain Places Its Ancestor in the HMG-Box Superfamily: Implication for Fungal Mating-Type Evolution
Source: PLoS One. 2010 Dec 8;5(12):e15199. doi: 10.1371/journal.pone.0015199 (PMC2999568; doi:10.1371/journal.pone.0015199)
Supplement: Table S2 — Top ten scoring with PHYRE for selected α1 domains. (DOC) [file pone.0015199.s004.doc]

| Query name (domain) | Fungus^a^ | Template^b^ (identity) | E-value^c^ | Estimated precision | Fold/PDB  descriptor |
| --- | --- | --- | --- | --- | --- |
| FMR1 (α1) | *P. anserina* | d1qrva (12 %) | 0.005 | 95 % | HMG |
|  |  | d2gzka2 (10 %) | 0.0072 | 95 % | HMG |
|  |  | d1i11a (14 %) | 0.008 | 95 % | HMG |
|  |  | c1wxlA (11 %) | 0.0082 | 95 % | dna binding protein |
|  |  | c1j47A (9%) | 0.0097 | 95 % | transcription/dna |
|  |  | d1wgfa (10 %) | 0.011 | 95 % | HMG |
|  |  | c1j46A (9%) | 0.012 | 95 % | Transcription/dna |
|  |  | d1j46a (9 %) | 0.012 | 95 % | HMG |
|  |  | d2lefa (11 %) | 0.012 | 95 % | HMG |
|  |  | d1k99a (7 %) | 0.012 | 95 % | HMG |
| mat A-1 (α1) | *N. crassa* | d1qrva (11 %) | 0.028 | 95 % | HMG |
|  |  | d2gzka2 (8 %) | 0.046 | 95 % | HMG |
|  |  | d1i11a (10 %) | 0.046 | 95 % | HMG |
|  |  | c1j47A (7 %) | 0.052 | 95 % | transcription /dna |
|  |  | c1wxlA (10 %) | 0.055 | 95 % | dna binding protein |
|  |  | d1k99a (7%) | 0.063 | 95 % | HMG |
|  |  | d1wgfa (9 %) | 0.065 | 95 % | HMG |
|  |  | c2yulA (10 %) | 0.067 | 95 % | transcription |
|  |  | d2lefa (7 %) | 0.069 | 95 % | HMG |
|  |  | d1gt0d (9 %) | 0.078 | 95 % | HMG |
| MAT1-1-1 (α1) | *C. heterostrophus* | d1qrva (15 %) | 0.0013 | 95 % | HMG |
|  |  | c1wxlA (14 %) | 0.002 | 95 % | dna binding protein |
|  |  | d2lefa (11 %) | 0.0035 | 95 % | HMG |
|  |  | d2gzka2 (11 %) | 0.0035 | 95 % | HMG |
|  |  | d1wgfa (8 %) | 0.0036 | 95 % | HMG |
|  |  | d1i11a (14 %) | 0.0038 | 95 % | HMG |
|  |  | d2gzka1 (12 %) | 0.0046 | 95 % | HMG |
|  |  | c1j47A (10 %) | 0.005 | 95 % | transcription/dna |
|  |  | c1wz6A (12 %) | 0.0051 | 95 % | transcription |
|  |  | d1j3da (10 %) | 0.0051 | 95 % | HMG |

^a^ For complete names and accession numbers, see Table S4.

^b^ Highest scoring template to the query. Templates are known structures from the PHYRE fold library. The percentage sequence identity between the query and template is displayed in brackets. This is calculated relative to the shortest sequence.

^C^ likelihood of structural homology.
