# Supplementary material for: Tracing the Origin of the Fungal α1 Domain Places Its Ancestor in the HMG-Box Superfamily: Implication for Fungal Mating-Type Evolution
Source: PLoS One. 2010 Dec 8;5(12):e15199. doi: 10.1371/journal.pone.0015199 (PMC2999568; doi:10.1371/journal.pone.0015199)
Supplement: Table S3 — Color scheme used for Jalview. (DOC) [file pone.0015199.s005.doc]

| Residue at position^a^ | Applied color | Rules for color (minimum %, residue group)^b^ |
| --- | --- | --- |
| A, I, L, M, F, W, V | BLUE | (+ 60%, WLVIMAFCHP) |
| R, K | RED | (+ 60%, KR), (+ 80%, K, R, Q) |
| N | GREEN | (+ 50%, N), (+ 85%, N, Y) |
| C | BLUE | (+ 60%, WLVIMAFCHP) |
| C | PINK | (100%, C) |
| Q | GREEN | (+ 60%, KR), (+ 50%, QE), (+ 85%, Q, E, K, R) |
| E | MAGENTA | (+ 60%, KR), (+ 50%, QE), (+ 85%, E, Q, D) |
| D | MAGENTA | (+ 60%, KR), (+ 85%, K, R, Q), (+ 50%, ED) |
| G | ORANGE | (+ 0%, G) |
| H, Y | CYAN | (+ 60%, WLVIMAFCHP), (+ 85% W, Y, A, C, P, Q, F, H, I, L, M, V) |
| P | YELLOW | (+ 0%, P) |
| S, T | GREEN | (+ 60%, WLVIMAFCHP), (+ 50%, TS), (+ 85%, S, T) |

^a^ if more than one residue is specified, the rules applied to each of these residues.

^b^ if a group of residues is concatenated together, such as 'WLVIMAFCHP', then any combination of these residues in total must meet or exceed the given percentage for the colour to be applied. For residues or residue groups separated by commas (*e*. *g*. W, Y, A, C, P, Q, F, H, I, L, M, V) at least one of these must by itself exceed the percentage.
