# Supplementary material for: Tracing the Origin of the Fungal α1 Domain Places Its Ancestor in the HMG-Box Superfamily: Implication for Fungal Mating-Type Evolution
Source: PLoS One. 2010 Dec 8;5(12):e15199. doi: 10.1371/journal.pone.0015199 (PMC2999568; doi:10.1371/journal.pone.0015199)
Supplement: Table S4 — Accession numbers for proteins of Figure 4 , Table 1 and Table S2. (DOC) [file pone.0015199.s006.doc]

| Protein name | Domain | Organism | Accession number |
| --- | --- | --- | --- |
| mSOX2 | SOX_HMG | *Mus musculus* | NP_035573 |
| MAT1-2-1/FPR1 | MATA_HMG | *Podospora anserina* | CAA45520 |
| MAT1-2-1/mat a-1 | MATA_HMG | *Neurospora crassa* | AAA33598 |
| MAT1-2-1 | MATA_HMG | *Cochliobolus heterostrophus* | CAA48464 |
| MAT1-2-1 | MATA_HMG | *Mycosphaerella graminicola* | AAL30836 |
| MAT1-1-3/SMR2 | MATA_HMG | *Podospora anserina* | CAA52051 |
| MAT1-1-3/mat A-3 | MATA_HMG | *Neurospora crassa* | AAC37476 |
| MAT1-1-3 | MATA_HMG | *Gibberella zeae* | AAG42812 |
| MAT1-1-3/phb1 | MATA_HMG | *Pyrenopeziza brassicae* | CAA06846 |
| MAT1-1-1/FMR1 | α1 | *Podospora anserina* | CAA45519 |
| MAT1-1-1/mat A-1 | α1 | *Neurospora crassa* | AAC37478 |
| SMTA-1 | α1 | *Sordaria macrospora* | CAA71623 |
| MAT1-1-1 | α1 | *Magnaporthe oryzae* | BAC65087 |
| MAT1-1-1 | α1 | *Cryphonectria parasitica* | AAK83346 |
| MAT1-1-1 | α1 | *Diaporthe sp G* | BAE93756 |
| MAT1-1-1 | α1 | *Gibberella fujikuroi* | AAC71055 |
| MAT1-1-1 | α1 | *Gibberella zeae* | AAG42809 |
| MAT1-1-1/pad1 | α1 | *Pyrenopeziza brassicae* | CAA06844 |
| MAT1-1-1 | α1 | *Aspergillus fumigatus* | AAX83122 |
| MAT1-1/MATB | α1 | *Aspergillus nidulans* | EAA63189 |
| MAT1-1-1 | α1 | *Histoplasma capsulatum* | ABO87596 |
| MAT1-1-1 | α1 | *Cochliobolus heterostrophus* | CAA48465 |
| MAT1-1-1 | α1 | *Mycosphaerella graminicola* | AAL30838 |
| Matα1p | α1 | *Saccharomyces cerevisiae* | EDN62161.1 |
| Pc | α1 | *Schizosaccharomyces pombe* | P10841 |
